# Dynamics of a 3D Piezo-Magneto-Elastic Energy Harvester with Axisymmetric Multi-Stability

**DOI:** 10.3390/mi15070906

**Published:** 2024-07-12

**Authors:** Grzegorz Litak, Mariusz Klimek, Abhijeet M. Giri, Piotr Wolszczak

**Affiliations:** Faculty of Mechanical Engineering, Lublin University of Technology, Nadbystrzycka 36, 20-618 Lublin, Poland; m.klimek@pollub.pl (M.K.); a.giri@pollub.pl (A.M.G.); p.wolszczak@pollub.pl (P.W.)

**Keywords:** piezoelectric, energy harvesting, nonlinear vibrations, axisymmetric, multi-stable

## Abstract

In this investigation, a three-dimensional (3D) axisymmetric potential well-based nonlinear piezoelectric energy harvester is proposed to increase the broadband frequency response under low-strength planar external excitation. Here, a two-dimensional (2D) planar bi-stable Duffing potential is generalized into three dimensions by utilizing axial symmetry. The resulting axisymmetric potential well has infinitely many stable equilibria and one unstable equilibria at the highest point of the potential barrier for this cantilevered oscillator. Dynamics of such a 3D piezoelectric harvester with axisymmetric multi-stability are studied under planar circular excitation motion. Bifurcations of average power harvested from the two pairs of piezoelectric patches are presented against the frequency variation. The results show the presence of several branches of large-amplitude cross-well type period-1 and subharmonic solutions. Subharmonics involved in such responses are verified from the Fourier spectra of the solutions. The identified subharmonic solutions perform interesting patterns of curvilinear oscillations, which do not cross the potential barrier through its highest point. These solutions can completely or partially avoid the climbing of the potential barrier, thereby requiring low input excitation energy for barrier crossing. The influence of excitation amplitude on the bifurcations of normalized power is also investigated. Through multiple solution branches of subharmonic solutions, producing comparable power to the period-1 branch, broadband frequency response characteristics of such a 3D axisymmetically multi-stable harvester are highlighted.

## 1. Introduction

Piezoelectric vibration energy harvesting has been known for many years [1,2,3,4]. The corresponding device often consists of a spring mass resonator and piezoelectric transducer. Usually, the frame with a resonator is excited kinematically by the ambient conditions or tested in the laboratory by inducing harmonic vibrations in a range of frequencies (frequency sweep) [5]. A piezoelectric transducer has the form of the layer placed along the most flexible part of the cantilever beam. The associated electrodes also connect it to the electrical circuit providing the electromotive force induced by the beam deformation. Piezoelectric transducers have a relatively high density of energy, enabling effective miniaturization. Such a system works well in the resonance region of a resonator structure. However, for ambient conditions (induced by wind, sea waves, and vehicle traffic) it is not sufficient due to variable working conditions [6]. Obviously, it is not possible to adjust the resonance conditions for various amplitudes and frequencies of ambient vibration sources. To overcome this limitation, a nonlinear resonator was proposed [7,8,9,10]. The effects of nonlinearities were summarized in the review presented in [11]. This work discussed a single resonance region, whereas a nonlinear system can possess several internal resonance regimes. Later, more attention was placed on subharmonic solutions [12,13,14,15,16,17,18], enabling multiple-cycle accumulation of energy on the input of the device to obtain a single cycle of response output. This procedure adapts the nonlinear system property of frequency transformation, namely, an output frequency is changed with respect to the input excitation frequency. In nonlinear systems, output response frequency is a sub-multiple (1/*n*) value of the input excitation frequency for a period-*n* solution. It should be noted that the response or transmission frequency ranges increase rapidly once multiple solutions appear together with various resonance areas [19,20,21]. The alternative way to multiple solutions is to increase the degree of freedom of the beam resonator to cover the increased number of natural frequencies in the transmission frequency interval [22,23].

It is also important to discuss about the possible advantages of the multi-stable equilibria positions. A 2D planar bi-stable potential well-based harvester can manifest large amplitude cross-well oscillations, which induce larger strains in the piezoelectric patches, resulting in higher output voltages. Further, several multi-stable potential-based harvesters, such as tri-stable [5], quad-stable [24], and even penta-stable [25] potential wells, were investigated. These and some other studies [26] have revealed the advantages of multi-stable equilibria positions which enable the large orbit oscillations spanning over these equilibria, and thereby inducing higher strains and voltages in the piezoelectric patches. However, having stable equilibria quite far away from the central zero equilibrium (for example tri-stable, quad-stable, or penta-stable) also induces strains which are higher than the allowable elastic limit for the piezoelectric patches [27]. In such cases, having multi-stable equilibria at the same radial distance around the central zero equilibrium could be beneficial for limiting the strains to a safe value. Therefore, investigating such a 3D axisymmetric multi-stable potential well-based system is quite promising in this aspect.

In this paper, we follow the multi-stability concept induced by the nonlinearity with a simultaneous extension of degrees of freedom resulting from the generalization of the configuration space from two to three dimensions. Here, referring to the analogy of a spherical pendulum from a planar one, a simple 2D bi-stable cantilever beam energy harvester is generalized to 3D by using the axial symmetry.

The discussion presented about this investigation is arranged as follows. In Section 2, we provide the description of the mathematical model which is based on the generalization of 2D planar Duffing-type bi-stable potential well. Model parameters used for simulating the dynamics of the axisymmetrically multi-stable system are mentioned in Section 2. Planar circular excitation parameters and the high and low energy-state initial conditions used to reveal multiple solution branches are also mentioned. Results of the numerical simulations are discussed in Section 3, which include the bifurcations of average power, trajectories of selected solutions from various branches, and their Fourier spectra (Section 3.1). Influence of the excitation amplitude on the bifurcations of average power is discussed in Section 3.2. In Section 4, concluding remarks about this investigation are presented.

## 2. Description of the Model

**Figure 1:**
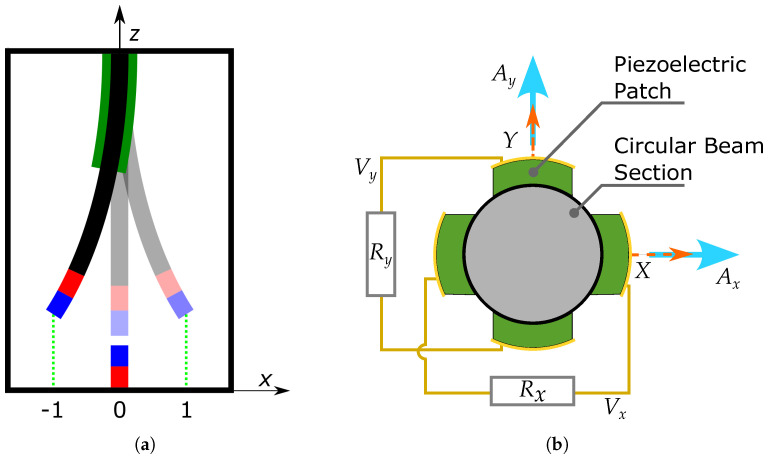
(**a**) Schematic front view of the system under consideration in the xz-plane. The vertical circular elastic beam (shown in black), tip and base magnets (shown in red and blue), and piezoelectric material patches (shown in green) are shown in the configuration. Stable and unstable equilibrium positions are shown at x=±1 and x=0, respectively. (**b**) Schematic top view presents the arrangement of the two pairs of piezoelectric patches, facing *x*- and *y*-directions in the xy-plane. External accelerations $A_{x}$ and $A_{y}$ are depicted using light blue-colored arrows.

**Figure 2:**
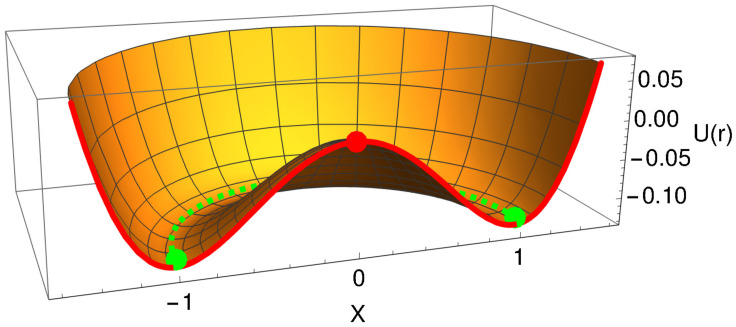
Axisymmetric configuration of the potential energy function U(r) (in the dimensionless model) is shown with $k_{1}=k_{2}=0.5$. Stable equilibrium positions are shown at x=±1 and along the green-colored circular trajectory, whereas an unstable equilibrium is shown at x=0 with a red dot.

The schematic views of the 3D axisymmetric energy harvesting system are presented in Figure 1. Here, Figure 1a shows the 2D xz-planar view of the axisymmetrical system. A cantilever beam with a circular cross-section carries a permanent magnet at the tip-point of the free end. Another such magnet is fixed to the frame, oppositely facing the tip magnet of the beam and interacting repulsively. Two pairs of the piezoelectric patches are mounted at the root of the cantilever beam, which form two separate electrical circuits as presented in Figure 1b. Such an arrangement of the piezoelectric patches was reported in [28] for a linear harvesting structure. In Figure 2, a 3D axisymmetrical potential well of the proposed system is shown. The locus of the multi-stable equilibria of the potential well, which results from the elastic and magnetic potential of the system, is shown by the green-colored circular trajectory.

The governing equations of motion for such a 3D beam structure have been previously reported in [29] by the authors. In [29], the extended Hamiltonian approach was followed and, using Euler–Lagrange’s equations, the governing electromechanical equations were derived. Here, we avoid the repetitive reporting and only present the derivation of the nonlinear restoring force corresponding to the 3D axisymmetric multi-stable potential well-based system. Also, we consider a circular excitation motion instead of the single-axis excitation considered in [29]. However, we follow the assumptions of small strain and small rotation for the deflections, thereby neglecting the nonlinear geometry effects in this work.

In the proposed mathematical model, $\vec{r}$ is the position vector of the tip point of the beam in a plane containing the tip of the beam at rest. $\vec{r}$ has the coordinates (x,y). The length of the vector is denoted by its magnitude $r=|\vec{r}|$. We assume that the potential energy U(r) is the function of the distance *r* only. Then, the restoring force of the system can be expressed as below.

(1) $$\vec{F}=F_{x}{\vec{e}}_{x}+F_{y}{\vec{e}}_{y}.$$

The restoring force $\vec{F}$ can be derived from the scalar potential function U(r) as follows.

(2) $$\vec{F}=-\nabla U=-\frac{\partial U}{\partial x}{\vec{e}}_{x}-\frac{\partial U}{\partial y}{\vec{e}}_{y}.$$

Thus, the components of $\vec{F}$ are given by

(3a) $$\begin{array}{cc} \; & F_{x}=-\frac{x}{\sqrt{x^{2}+y^{2}}}U^{\prime }\left(\sqrt{x^{2}+y^{2}}\right), \end{array}$$

(3b) $$\begin{array}{cc} \; & F_{y}=-\frac{y}{\sqrt{x^{2}+y^{2}}}U^{\prime }\left(\sqrt{x^{2}+y^{2}}\right), \end{array}$$

where the corresponding potential derivative regarding *r* reads as follows.

(4) $$U^{\prime }\left(\sqrt{x^{2}+y^{2}}\right)=\frac{dU(r)}{dr}.$$

In this work, we focus our attention on a two-dimensional double-well Duffing oscillator, i.e., the potential of the form considered in [7,9,15], and represented by the following equation.

(5) $$U\left(r\right)=-\frac{k_{1}}{2}r^{2}+\frac{k_{2}}{4}r^{4},$$

where $k_{1}$ represents the linear softening part of magnetic potential along with the elastic potential of the beam, and $k_{2}$ represents the cubic nonlinearity coefficient arising from magnetic potential. The nonlinear restoring force is derived using Equations in (3) and (4) for this well-known bi-stable Duffing potential represented by Equation (5) [30]. Use of the radial coordinate r(t) in this Duffing’s equation results in the generalization of planar bi-stable potential well into the 3D axisymmetric multi-stable potential well. The resulting 3D potential well U(r) is plotted in Figure 2 for the visualisation purpose.

The governing electromechanical Equations in (6), after substituting the $U^{\prime }\left(r\right)$ expression computed using Equation (5), represent the dynamic equilibrium of a 3D axisymmetrically multi-stable piezoelectric harvester system. These equations of motion are presented in the dimensionless form (see [7,9,15] for the 2D analog).

(6a) $$\begin{array}{cc} \ddot{x}+2\zeta \dot{x}+\frac{x}{\sqrt{x^{2}+y^{2}}}U^{\prime }\left(\sqrt{x^{2}+y^{2}}\right)-\chi_{x}v_{x} & =F_{x}^{ext}, \end{array}$$

(6b) $$\begin{array}{cc} \ddot{y}+2\zeta \dot{y}+\frac{y}{\sqrt{x^{2}+y^{2}}}U^{\prime }\left(\sqrt{x^{2}+y^{2}}\right)-\chi_{y}v_{y} & =F_{y}^{ext}, \end{array}$$

(6c) $$\begin{array}{cc} {\dot{v}}_{x}+\lambda_{x}v_{x}+\kappa_{x}\dot{x} & =0, \end{array}$$

(6d) $$\begin{array}{cc} {\dot{v}}_{y}+\lambda_{y}v_{y}+\kappa_{y}\dot{y} & =0, \end{array}$$

where ζ is a mechanical damping factor; κ and χ represent the forward and backward piezoelectric coupling coefficients, respectively; $F^{ext}$ denotes the inertial excitation forces; *v* denotes the voltage across the load resistances; λ∝1/RC is the reciprocal of the time constants of the equivalent electrical circuit, in which load resistance *R* and piezoelectric capacitance *C* are involved. In the governing Equations in (6), the subscripts *x* and *y* are associated with the above dimensionless system parameters in the respective *x*- and *y*-direction equations.

Interestingly, in such a 3D potential well, the number of stable equilibrium points has increased considerably compared to the planar bi-stable potential case. In the case of planar bi-stable potential, one unstable equilibrium at x=0 and two stable equilibria at x=±1 are observed. However, the situation is changed due to the axisymmetrical generalization of such a potential. The two stable equilibria have now transformed into infinitely many stable equilibria, which are shown by a green-colored circular trajectory in Figure 2. The unstable equilibrium point is preserved on the vertical axis of symmetry and is located at the highest point of the potential barrier.

Note that, as a result of such a generalization, infinitely many paths are now available for large cross-well type oscillations. All such paths can partially or completely avoid the entire climbing of the potential barrier, i.e., the highest point of potential barrier. In this way, the system has gained the additional rotating or curvilinear-type oscillation mode besides the usual planar oscillation mode known for the planar dynamical system. The appearance of these two modes would depend on the assumed initial conditions and excitation parameters of the beam shown in Figure 1a. Thus, since the 3D axisymmetric multi-stable potential configuration possesses many paths that can avoid the direct crossing of the potential barrier to perform a cross-well motion, it greatly improves the possibility of such a large amplitude oscillation, even at low strength excitation. This investigation is also aimed at revealing such cross-well motion paths requiring lower excitation energy. In case of the planar bi-stable system, all the cross-well oscillations require a compulsory crossing of the potential barrier through its highest point. The model parameters, excitation parameters, and initial conditions used for numerical simulations are discussed in the following subsection.

### Description of Model and Excitation Parameters

While performing numerical simulations, we consider the values of system parameters appearing in the Equations in (6) as ζ=0.01, $k_{1}=k_{2}=0.5$, $\chi_{x}=\chi_{y}=0.05$, $\lambda_{x}=\lambda_{y}=0.01$, and $\kappa_{x}=\kappa_{y}=0.5$. Such a choice of system parameters is based on the investigations performed in the previous work [31]. As these parameters are dimensionless, it allows us to model a 3D multi-stable system which is equivalent to the 2D planar bi-stable system investigated in [31]. Also, it can be verified that the governing Equations in (6) reduce to the equations of a 2D planar bi-stable system as reported in [31] if the excitation in the *y*-direction is neglected.

The components of excitation force representing a planar circular motion in the xy plane are computed as follows.

(7a) $$F_{x}^{ext}=0.183\omega^{2}\cos\left(\omega t\right),$$

(7b) $$F_{y}^{ext}=0.183\omega^{2}\sin\left(\omega t\right),$$

where the circular excitation frequency ω varies in the closed interval [0.1,4.0]. The following two initial conditions are also considered for observing the multiple solutions.

(8) $$x(0)=0,\;\mathrm{and}\;\dot{x}\left(0\right)=y\left(0\right)=\dot{y}\left(0\right)=v_{x}\left(0\right)=v_{y}\left(0\right)=0,$$

(9) $$x(0)=1,\;\mathrm{and}\;\dot{x}\left(0\right)=y\left(0\right)=\dot{y}\left(0\right)=v_{x}\left(0\right)=v_{y}\left(0\right)=0.$$

In the above initial conditions, the first set given by Equation (8) corresponds to a higher initial energy state, whereas the second set given by Equation (9) represents a lower energy state. Note that the external force indicates the inertial circular kinematic excitation of the frame (Figure 1) where $A_{x}=A_{y}=0.183\omega^{2}$ are the acceleration amplitudes. We investigate the system’s response for the above two sets of initial conditions staring from the potential equlibria.

By considering the above-mentioned system parameters, external forcing conditions, and sets of initial conditions, the governing Equations in (6) are solved numerically using the LSODA solver of the Mathematica (v13.2) software. Such a solver is capable of switching between the non-stiff and stiff methods of numerical differentiation as necessary. At each excitation frequency ω, the system is simulated for 1000 forcing cycles, each with a time period $t_{p}=2\pi /\omega$. Thus, total simulation time is computed as follows,

(10) $$t_{max}=1000\;\frac{2\pi }{\omega }.$$

The average power $\bar{P}$ of a response is then computed over the last n=20 excitation periods. For this, we considered the unit resistance values for $R_{x}=R_{y}=1$. The expression for computing the average power is written below.

(11) $$\bar{P}=\frac{1}{nt_{p}}\int_{\left(1000-n\right)t_{p}}^{t_{max}}\left(v_{x}^{2}+v_{y}^{2}\right)dt.$$

## 3. Numerical Simulations and Results

In order to study the dynamic characteristics of the proposed 3D axisymmetric potential-based system, governing Equations in (6) are numerically simulated. The numerical simulations are aimed at revealing the multiple solutions and their branches, the super- and sub-harmonics involved in building the response of the system, and the types of curvilinear oscillations. For such a revelation, we have chosen the high and low levels of external excitation amplitudes as well as high- and low-energy state initial conditions of the system. Under such excitation and initial conditions, we have computed and analyzed the bifurcations of average power over a range of excitation frequencies. In the following subsection, we have discussed these results using bifurcation diagrams of average power, planar oscillation trajectories of tip point, and Fast Fourier Transform (FFT) of oscillations.

### 3.1. Results and Discussion

**Figure 3:**
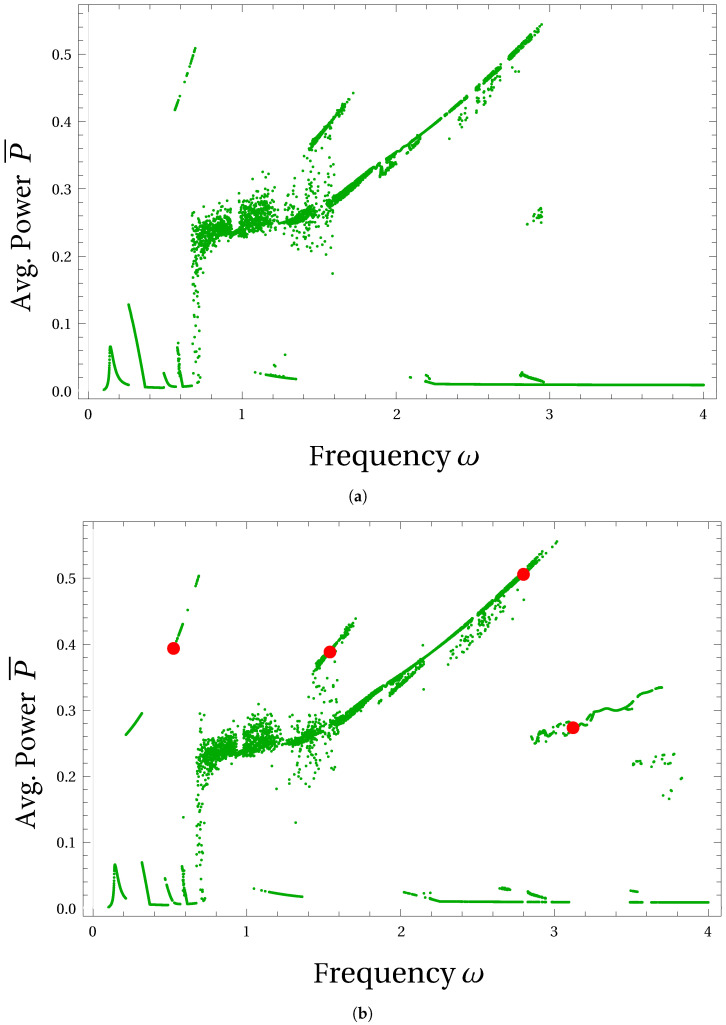
Bifurcations of the average power output $\bar{P}$ against the excitation frequency are shown here for the (**a**) low- and (**b**) high-energy state initial conditions given by Equations (8) and (9), respectively. Red-colored dots marked in (**b**) represent the periodic solution trajectories shown in Figure 4.

In Figure 3a and Figure 3b, the bifurcation diagram for average power is obtained with numerical frequency sweep simulations using the low- and high-energy state initial conditions, respectively. It can be observed that, just like the planar bi-stable oscillator, the 3D axisymmetric multi-stable harvester system also depicts high and low amplitude periodic solutions along with some chaotic solution regions. A region dominated by chaotic solutions can be observed over the dimensionless frequency range of [0.7,1.6]. For the low-energy state initial condition, high-amplitude cross-well type solutions appear only after ω=0.6. However, for the high-energy state initial condition, such cross-well solutions appear at excitation frequencies as low as ω≈0.2. Also, the high-amplitude periodic solution branch, appearing over a range ω=2.8to3.6 under the high-energy state initial condition, disappears under the low-energy state initial condition. Between ω=0.8to2.8, the observed high and low amplitude periodic solution branches are attainable by both high- and low-energy state initial conditions.

Higher power output branches are related to the inter-potential well (cross-well) motion of the mechanical resonator (Figure 1), while the lower branches, usually non-resonant, correspond to the single or intra-potential well motion of the resonator. In the second case, the oscillations can be associated with the rotational mode. In both diagrams of Figure 3, it is possible to find the narrow and broad character of branches. This is a feature of solution regularity. The narrow branch indicates the periodic solution while the broad non-periodic (presumably chaotic as in the planar system [15]) branch corresponds to a solution in the region nearby ω≈1.

**Figure 4:**
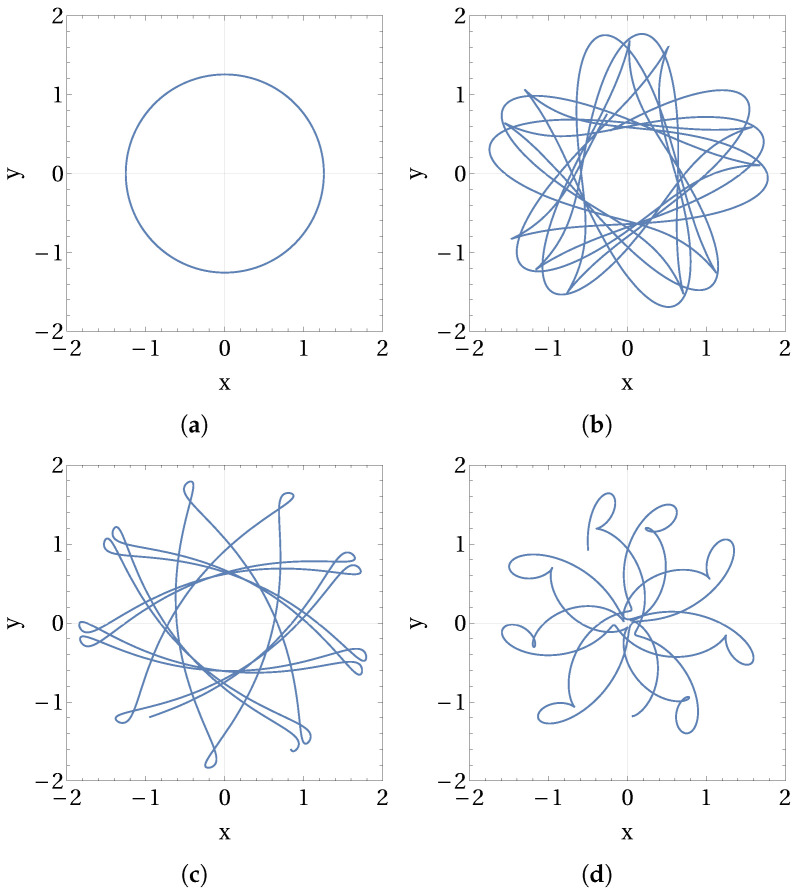
Trajectories of the curvilinear oscillations of the periodic responses are shown here. These trajectories are observed at the selected frequencies (**a**) ω=0.5235, (**b**) ω=1.5405, (**c**) ω=2.7985, and (**d**) ω=3.121 that are marked by red-colored dots in Figure 3b.

**Figure 5:**
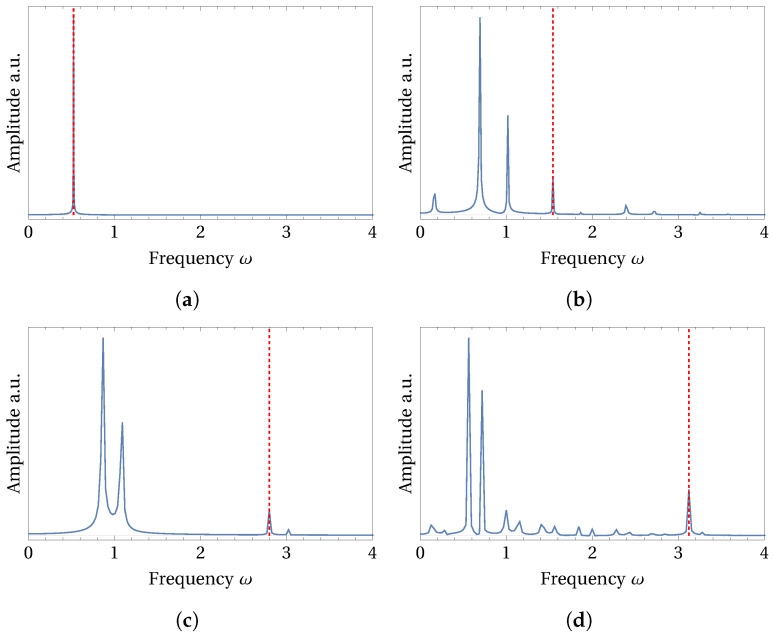
Fourier spectra (FFT) of the four periodic response trajectories shown in Figure 4a–d are shown here in (**a**–**d**), respectively. Red-colored dashed line marks the frequency of excitation. Subharmonic frequency peaks in the FFTs shown in (**b**–**d**) confirm the subharmonic nature of the periodic solutions shown in Figure 4a–d. (**a**) ω=0.5235, (**b**) ω=1.5405, (**c**) ω=2.7985, (**d**) ω=3.121.

Although there are many intra-well type small-amplitude solutions visible in Figure 3a,b throughout the frequency range, we have focused on the high-amplitude periodic solution branches that produce higher average power output. In Figure 3b, we marked four red-colored dots on the four high-amplitude branches of periodic solutions. These branches are also attainable under low-energy state initial conditions. Periodic responses at these four locations are further analyzed by observing the patterns of the curvilinear oscillations, which are shown in Figure 4. Note that, these four curvilinear trajectories are obtained under the external circular forcing motion described by the Equations in (7). In order to confirm the periodicity of these solutions and to identify the super- and sub-harmonics that dominate the response, we presented the FFTs of all the trajectories shown in Figure 4a–d in Figure 5a–d, respectively.

In Figure 4a, the circular shape of the response trajectory confirms that the response exactly follows the circular forcing motion, hinting at a large-amplitude period-1 solution. This fact is confirmed by observing the single peak appearing in the FFT of this solution shown in Figure 5a. The sole frequency peak appearing here also coincides with the excitation frequency marked by a red-colored dashed line in the FFT plot, confirming the period-1 harmonic solution. It can also be observed that such a period-1 response completely avoids the direct climbing or crossing of the potential barrier. Therefore, the power produced by the period-1 response shown in Figure 4a, although obtained at a lower frequency of ω=0.5235, is comparable with the solutions from other branches obtained at the higher frequencies.

Under the same circular forcing conditions, the periodic responses obtained from the other three branches produce dissimilar non-circular trajectories. The trajectories shown in Figure 4b–d partially climb the central potential barrier and then reach the opposite sides of the original positions. As the excitation frequency increases and takes the values ω= 1.5405, 2.7985, and 3.121, the number of passes made by these trajectories in crossing the potential barrier also increases. With the increasing excitation frequency, trajectories also trace mixed oscillations consisting of local intra-well loops along with the large cross-well loops. Such a trend can be observed in Figure 4b–d, which hints at the presence of additional harmonics in the responses. Such a prediction is confirmed by the additional frequency peaks present in the FFT of these solutions, as shown in Figure 5b–d. However, these additional peaks in FFT are located at lower frequencies than the excitation frequency peak marked by red-colored dashed lines in the respective solution plots. Hence, the response trajectories shown in Figure 4b–d belong to the subharmonic solutions category [16]. In Table 1, frequency locations of the additional subharmonic peaks in FFT are listed for the four periodic solutions shown in Figure 4. It can be noted that the average power produced by the above subharmonic solutions is on par with the period-1 solution shown by Figure 4a. However, such subharmonic solutions appear at higher excitation frequencies which supply more energy into the system compared to the period-1 response appearing at the lower excitation frequency. In this study, we have not investigated the basins of attraction for the observed solutions which could reveal their stability and probability of occurrence.

### 3.2. Influence of the Excitation Amplitude on the Average Power

**Figure 6:**
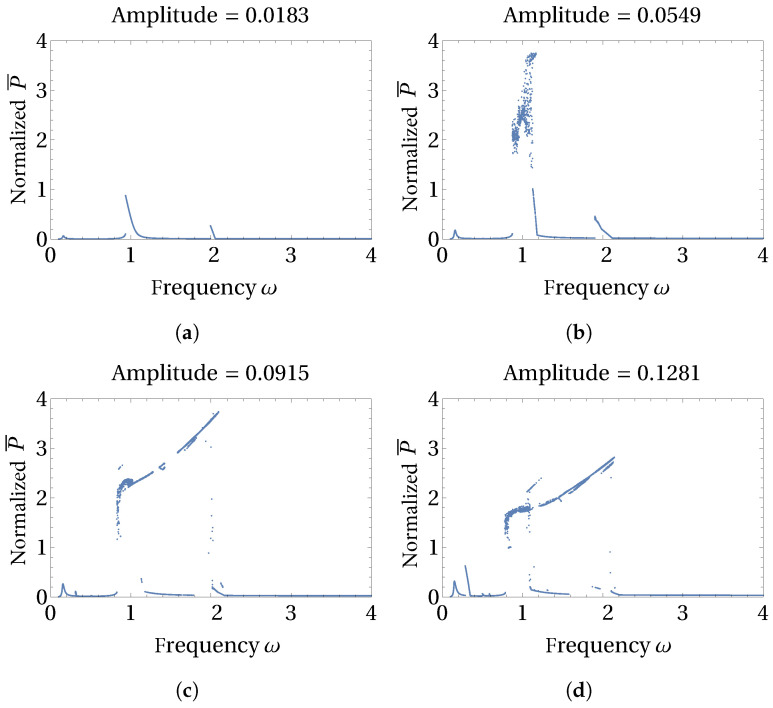
Influence of excitation amplitude on the bifurcations of normalized average power are shown here. Low-strength excitation amplitude levels of (**a**) 0.0183, (**b**) 0.0549, (**c**) 0.0915, and (**d**) 0.1281 are chosen to reveal the influence of low excitation amplitude levels.

**Figure 7:**
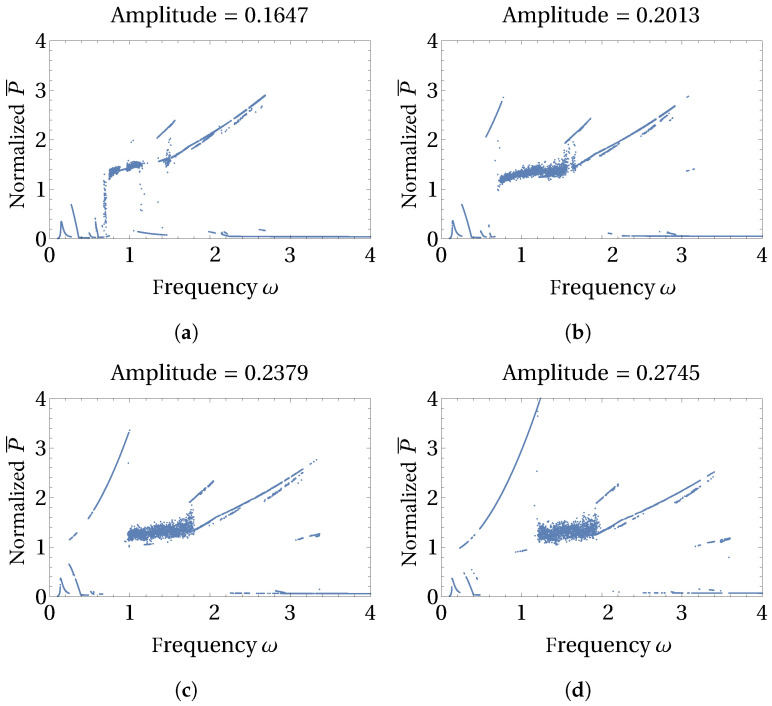
Influence of excitation amplitude on the bifurcations of normalized average power are shown here. Relatively higher levels of excitation amplitudes with values (**a**) 0.1647, (**b**) 0.2013, (**c**) 0.2379, and (**d**) 0.2745 are chosen to reveal the influence of high excitation amplitude levels.

The appearance of multiple periodic and chaotic solutions of the 3D axisymmetric system highly depends on the input energy provided to the system through the amplitude and frequency of excitation as well as the initial conditions. Through Figure 6 and Figure 7, we present the influence of low and high levels of excitation amplitudes, respectively, on the multiple solutions and normalized power produced by these solutions. We selected a low-energy state initial condition for studying the influence of excitation amplitude, and the average power is normalized to obtain the average power produced per unit of base displacement amplitude. At the excitation amplitude of 0.0183 (see Figure 6a), which is one order less than the value used for previous simulations, only small-amplitude intra-well type solutions exist. The amplitude of intra-well resonance peaks of such small-amplitude solutions increases with the increasing excitation amplitude (Figure 6b), and large-amplitude cross-well solution branches emanate when the excitation amplitude reaches 0.0915 (see Figure 6c). Further increase in excitation amplitude to a value of 0.1281 promotes the manifestation of a subharmonic solution branch as observed in Figure 6d.

When the higher levels of excitation amplitudes are chosen (see Figure 7), several subharmonic solution branches can be observed along with the region of chaotic solutions. The high-amplitude period-1 solution branch (Figure 4a) does not appear until excitation amplitude reaches 0.183. The amplitude of such a period-1 solution tracing a circular oscillation path grows sharply when the excitation amplitude levels reach 0.2379 and beyond (see Figure 7c,d). Frequency ranges of the subharmonic solution branches also extend as the higher levels of excitation amplitude are employed. Another observation regarding the stability of the period-1 and subharmonic solution branches is prominently visible in Figure 7. These branches are more continuous at higher levels of excitation amplitude, which hints at increased stability and robustness of the solutions. This observation is consistent with the observations reported in [16] about the stability of period-*n* solutions.

## 4. Conclusions

In this study, a 3D axisymmetric multi-stable potential well-based piezoelectric energy harvester is realized by generalizing the 2D planar bi-stable Duffing potential. In the resulting 3D potential well, infinitely many stable equilibria are generated due to axial symmetry. The influence of low- and high-energy state initial conditions is verified, and it is found that high-energy state initial conditions provide additional energy to attain the period-1 and subharmonic solution branches over a longer range of excitation frequencies. Chaotic solutions with a limit of a large number of excitation periods with respect to the single period of system response were observed. This observation regarding chaotic solutions is consistent with the subharmonic response scenario.

Numerical frequency sweep simulations performed under constant amplitude of excitation highlight the presence of additional large-amplitude subharmonic solution branches that produce equivalent power as produced by period-1 solutions. The presence of such solutions over a range which is four times the linear resonance frequency (i.e., ω=1) confirm the broadband response characteristics of this 3D multi-stable nonlinear piezoelectric harvester. Also, unlike in the case of the planar bi-stable oscillator, which requires compulsory crossing of the potential barrier through its highest point, many period-*n* responses of this 3D axisymmetric oscillator partially or completely avoid the climbing or crossing of the entire potential barrier. In such cases, even the low-strength excitation and initial conditions can promote the cross-well type large-amplitude oscillations which help to produce more power output. Analyzing this interesting aspect from the perspective of input energy requirements for such solutions is considered a future task for our investigation. Furthermore, such numerical simulations will also be verified using the parameters and feedback from our planned experiments.

## Figures and Tables

**Table 1:** Location of the excitation and subharmonic frequency peaks appearing in the FFTs shown in Figure 5.

Excitation Frequency	Frequencies of the Harmonics in FFT
0.5235	0.5235
1.5405	0.169455, 0.693225, 1.01673, 1.5405
2.7985	0.867535, 1.09142, 2.7985
3.1210	0.56178, 0.71783, 0.99872, 3.1210

## Data Availability

The data presented in this study are available on request from the corresponding author.
